# A review of the pathophysiology and the role of ion channels on bronchial asthma

**DOI:** 10.3389/fphar.2023.1236550

**Published:** 2023-09-28

**Authors:** Indyra Alencar Duarte Figueiredo, Sarah Rebeca Dantas Ferreira, Jayne Muniz Fernandes, Bagnólia Araújo da Silva, Luiz Henrique César Vasconcelos, Fabiana de Andrade Cavalcante

**Affiliations:** ^1^ Programa de Pós-graduação em Produtos Naturais e Sintéticos Bioativos, Centro de Ciências da Saúde, Universidade Federal da Paraíba, João Pessoa, Paraíba, Brazil; ^2^ Graduação em Farmácia, Departamento de Ciências Farmacêuticas, Centro de Ciências da Saúde, Universidade Federal da Paraíba, João Pessoa, Paraíba, Brazil; ^3^ Departamento de Ciências Farmacêuticas, Centro de Ciências da Saúde, Universidade Federal da Paraíba, João Pessoa, Paraíba, Brazil; ^4^ Departamento de Fisiologia e Patologia, Centro de Ciências da Saúde, Universidade Federal da Paraíba, João Pessoa, Paraíba, Brazil

**Keywords:** asthma, TRP channels, ORAI channels, KCa channels, TMEM16A channel, CFTR, Piezo1 channel, P2X receptor

## Abstract

Asthma is one of the main non-communicable chronic diseases and affects a huge portion of the population. It is a multifactorial disease, classified into several phenotypes, being the allergic the most frequent. The pathophysiological mechanism of asthma involves a Th2-type immune response, with high concentrations of allergen-specific immunoglobulin E, eosinophilia, hyperreactivity and airway remodeling. These mechanisms are orchestrated by intracellular signaling from effector cells, such as lymphocytes and eosinophils. Ion channels play a fundamental role in maintaining the inflammatory response on asthma. In particular, transient receptor potential (TRP), stock-operated Ca^2+^ channels (SOCs), Ca^2+^-activated K^+^ channels (IK_Ca_ and BK_Ca_), calcium-activated chloride channel (TMEM16A), cystic fibrosis transmembrane conductance regulator (CFTR), piezo-type mechanosensitive ion channel component 1 (PIEZO1) and purinergic P2X receptor (P2X). The recognition of the participation of these channels in the pathological process of asthma is important, as they become pharmacological targets for the discovery of new drugs and/or pharmacological tools that effectively help the pharmacotherapeutic follow-up of this disease, as well as the more specific mechanisms involved in worsening asthma.

## 1 Introduction

Characterized by variable respiratory symptoms, asthma can be defined as a chronic inflammatory disease, and the subject might present wheezing, increased chest pressure, cough, shortness of breath and limited expiratory flow. Asthma also can be triggered by several factors, including the exposure to allergens or irritants, respiratory infections, climate change or physical exercise. In the last decade approximately 260 million individuals were reported worldwide, causing 455,000 deaths and observing an increase in the prevalence of bronchial asthma (Who, 2023; Gina, 2022).

This condition involves a collaboration between various cells of the innate and adaptive immune system, alongside epithelial cells, to enhance bronchial hyperreactivity. It is characterized by an hyperresponsiveness of smooth muscle cells in the bronchial lumen to non-specific triggers, like cold air and physical exertion. Additionally, it leads to excessive mucus production, remodeling of the airway walls, and constriction of the passageways through which air flows (Hammad and Lambrecht, 2021).

At present, the primary approach to treating asthma primarily revolves around the utilization of bronchodilators. These include cholinergic antagonists, β-adrenergic agonists, antileukotrienes, and non-specific phosphodiesterase inhibitors. Alternatively, treatment may also involve the suppression of inflammation, wherein glucocorticoids serve as the primary option (Gina, 2022). These strategies help to control asthma symptoms, however they have several side effects, such as glaucoma, metabolic imbalance, tachycardia, dry mouth, mydriasis, constipation and immunosuppression (Walsh, 2005; Donohue et al., 2019). Therefore, studies to better elucidate possible pharmacological targets, such as ion channels, open the possibility of looking forward to new anti-asthmatic therapies. Therefore, this review will provide scientific support about ion channels that play an important role in the pathophysiology of asthma. These specialized membrane proteins regulate the flow of ions across cell membranes, thereby influencing various cellular processes. In asthma, specific ion channels participate in the dysregulation of bronchoconstriction, airway inflammation, and mucus production (Valverde et al., 2011; Bartoszewski et al., 2017; Pelletier and Savignac, 2018).

## 2 Epidemiological data on asthma

In 2019, approximately 262 million individuals worldwide were affected by asthma. The prevalence of this condition continues to rise, with projections indicating an expected increase of over 100 million people by the year 2025 (Global Asthma Network, 2018; Tohidinik et al., 2019; Who, 2023). Epidemiological data show that over the decades there has been almost double the number of cases (Antó, 2012).

Asthma stands as the most prevalent chronic disease among children, impacting approximately 24% of this population. Moreover, it affects around 19% of teenagers (Who, 2023). From a global perspective, the financial burden of asthma surpasses the combined costs associated with tuberculosis and HIV/AIDS (ASBAI, 2023). Despite the concerning statistics, asthma remains underdiagnosed. The World Health Survey (WHS) estimated that merely 4.3% of asthma cases were diagnosed globally, showcasing substantial variations of up to 21-fold between countries (To et al., 2012). These discrepancies in asthma prevalence emphasize the importance of conducting further studies to enable comparisons and facilitate the planning of appropriate interventions.

Brazil holds the 8th position globally in terms of asthma prevalence. In 2015, there were 383,000 reported deaths and according to the *Sistema Único de Saúde* (SUS), among the cases of hospitalization, asthma attacks ranked as the fourth type of cause (Cardoso et al., 2017).

## 3 Etiological factors

Asthma is a chronic inflammatory disease and its development involves environmental and genetic factors (Hammad and Lambrecht, 2021). This disease is characterized by its different phenotypes, which have distinct pathophysiological mechanisms and clinical manifestations, making diagnosis difficult (Moore et al., 2010). The possibility of developing and progressing asthma in genetically susceptible individuals is determined by various factors, including aspects related to immunological maturation and the timing of exposure to local agents, particularly during the childhood. These factors play an essential role in the likelihood of developing asthma in susceptible individuals (Busse and Lemanske, 2001).

**FIGURE 1:**
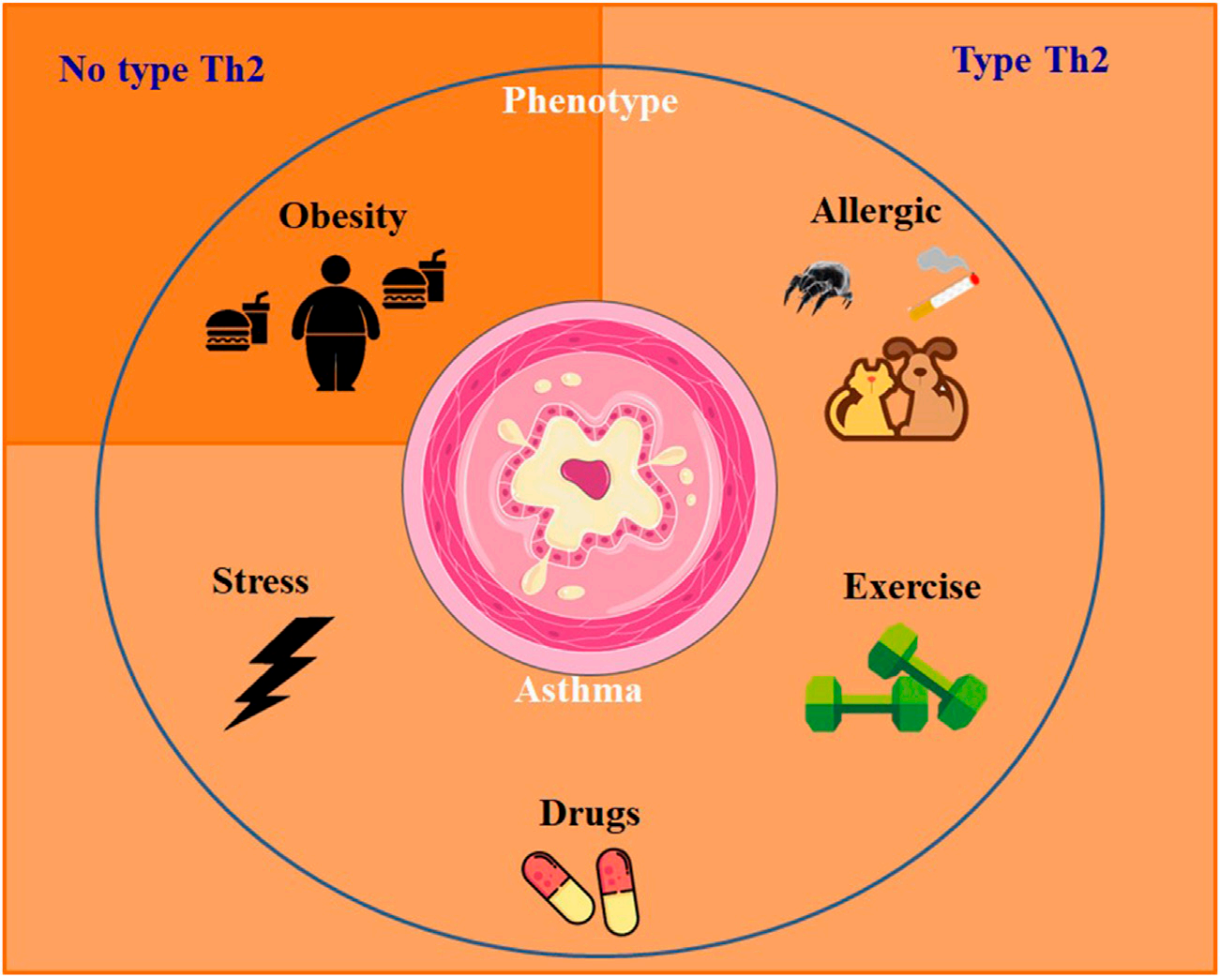
Mainly asthma phenotypes and inflammatory response profile.

Genetic factors, several comorbidities and risk factors are involved on the development of asthma, such as in individuals with severe or uncontrolled asthma. Exposure to dust mites, pet dander, cockroaches and *Aspergillus* genus fungi are notable risk factors. Other risk factors include smoking, physical exercise, and comorbidities such as obesity, respiratory infections, psychiatric illnesses, stress, rhinosinusitis, and non-steroidal anti-inflammatory drugs (Figure 1). These factors can collaborate to the worsening of asthma symptoms and should be carefully considered in the management and treatment of individuals with asthma.

Recent studies have placed increasing emphasis on the heterogeneity of asthma, leading to the recognition that asthma comprises multiple phenotypes. These phenotypes represent distinct groups with specific characteristics and varied expressions of the disease (Wenzel, 2012). Numerous phenotypes of asthma were identified, nevertheless the most common is allergic asthma. This particular phenotype typically emerges during childhood and is frequently linked to a family genetic conditions of allergy like rhinitis, eczema or allergies to food or medications. Identifying the allergic asthma phenotype is crucial for implementing tailored management strategies that involve allergen avoidance, immunotherapy, and targeted medication interventions to address the specific allergic triggers involved (Bel, 2004). Other phenotypes include the non-allergic asthma, aggravated by obesity and exercise (Moore et al., 2010; Wenzel, 2012).

### 3.1 Pathophysiology of asthma

Asthma is a chronic inflammatory disease of the airways, which causes bronchial hyperresponsiveness, tissue remodeling, lumen narrowing and excessive mucus production, especially by the action of immune system cells in association with epithelial cells (Hammad and Lambrecht, 2021). Since the immunity can be classified into type 1 and 2 T helper, asthma was regarded as a pathological process Th2 profile which is strongly linked to hypersensitivity reactions of type I (allergy). In fact, data from several studies suggest that most asthma cases fit this traditional view (Moore et al., 2010; Siroux et al., 2011).

In the lungs of several patients with allergic asthma, mainly Th2 profile cells are found (Robinson et al., 1992), which are responsible for the generation of cytokines that stimulate the synthesis of immunoglobulin E (IgE) antibodies by modulating IL-4. Additionally, IL-13, IL-5 and IL-9 are also important in this process, aiding in the recruitment of eosinophils and mast cells, culminating in increased bronchial hyperresponsiveness - an essential feature of asthma (Holgate and Polosa, 2008).

Activation of adaptive immunity depends on antigen-presenting cells (APCs), such as dendritic cells (DCs), whose role is crucial in triggering the Th2 response in asthma. DCs perform this action by presenting antigen through the major histocompatibility complex type II (MHC II) molecule. In the lung, DCs are distributed throughout the airways, vasculature, pleura, lung interstitial space, and bronchial lymph nodes. Their strategic positioning allows them to efficiently capture and process antigens, initiating immune responses (Lambrecht et al., 2019).

In allergic patients, whose immune response is directed towards the Th2 profile, activation of the T cell-specific trans-action transcription factor (GATA-3) occurs and characteristic cytokines are synthesized and secreted, such as IL-4, IL-5 and IL-13. The cytokines responsible for the isotype switch from IgG to IgE by plasma cells are IL-4 and IL-13, especially (Romagnani, 2000), in addition to the structural remodeling observed in chronic asthma. These structural changes include metaplasia of epithelial cells into Goblet cells and subepithelial fibrosis, mucus hypersecretion, fibroblasts differentiation to myofibroblasts and smooth muscle hyperplasia (Doucet et al., 1998).

Recruitment of eosinophils to lung tissue is fundamentally carried out by IL-5. The development of eosinophils in the bone marrow and their subsequent migration to the pulmonary mucosa and interstitium are made possible by the action of eotaxins 1, 2 and 3 (CCL11, CCL24 and CCL26, respectively). The action of these chemokines occurs by activation of the chemokine receptor CCR3, whose location occurs numerously in eosinophils, leading to their targeted recruitment to inflamed lung tissue (Ponath et al., 1996). Once stimulated, eosinophils assume a crucial pro-inflammatory role by producing cysteinyl-leukotrienes (Cys-LTs), along with additional Th2 cytokines. These inflammatory mediators contribute to the multiplication of the inflammatory response and exacerbate the allergic cascade in asthma (Akuthota et al., 2008). Products derived from eosinophils, such as eosinophilic peroxidase (EPO), major basic protein (MBP) and eosinophilic cationic protein (ECP), directly cause tissue damage and bronchial hyperresponsiveness, in addition to activating adaptive immunity through effects on DCs (Coyle et al., 1995; Chu et al., 2014).

Other important cells in allergic asthma are mast cells, which express type I Fcε receptors (FcεRI) and reside near the mucosal surface and in blood vessels. Local factors influence the maturation of these cells, mainly stem cell factor (SCF) and IL-3 (Gurish and Austen, 2012). Immediate hypersensitivity reactions can be initiated by mast cells upon subsequent allergen challenges. They degranulate in response to signals from both the adaptive immune system, through antigen cross-linking to IgEs bound to FcεRI receptors, and the innate immune system, triggered by Toll-like receptor agonists or cytokines like IL-33 (Stone et al., 2006; Silver et al., 2010).

This degranulation process stimulates the release of several inflammatory mediators, including histamine, prostaglandins, leukotrienes, and cytokines. Once activated, these cells release histamine, Cys-LTs, prostaglandin D_2_ (PGD_2_), platelet activating factor (PAF), in addition to cytokines and chemokines (Galli et al., 2005; Barrett and Austen, 2009). Basophils, which are circulating granulocytes, also express FcεRI and amplifying immediate hypersensitivity responses. The contribution to the allergic cascade also occurs by IL-4 production (Yoshimoto et al., 2009). Mast cell granules store previously synthesized cytokines, including IL-5, IL-6 (Bradding et al., 1993), IL-4 (Reed et al., 1995), tumor necrosis factor (TNF) (Gordon and Galli, 1990), as well as transforming growth factor β (TGF-β) (Lindstedt et al., 2001) and vascular endothelial growth factor (VEGF) (Boesiger et al., 1998; Grutzkau et al., 1998).

**FIGURE 2:**
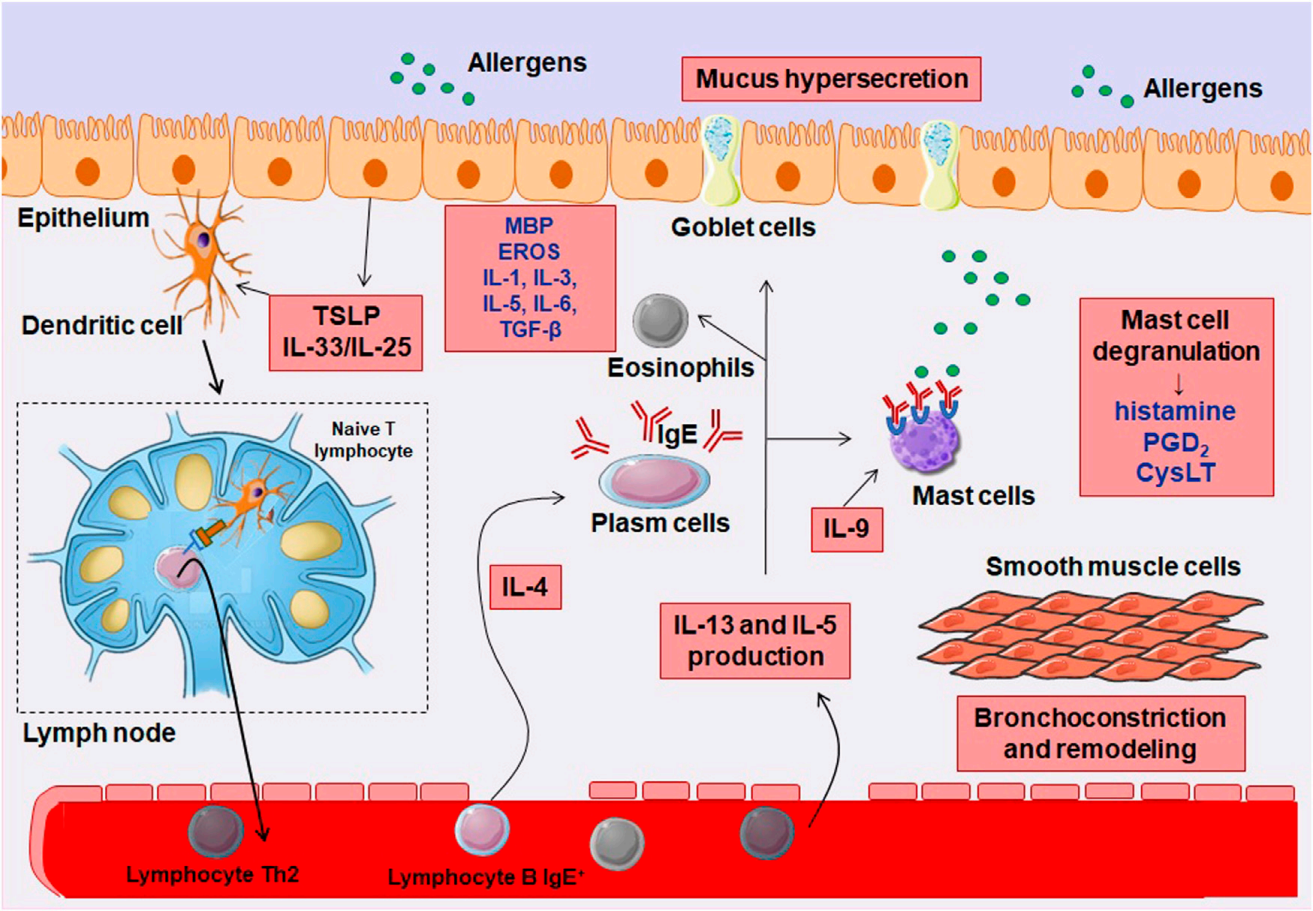
Inflammatory pathophysiology of allergic asthma. In the presence of an allergen, there is the release of cytokines from the epithelium, particularly IL-25 and IL-33, which induces co-stimulatory molecule of T lymphocytes in dendritic cells (DCs), which act to promote T cell survival and expansion. DCs are mobilized to local lymph nodes, where they present the antigenic peptide to naive CD^4+^ T cells, activating and converting them to a competent IL-4-producing state. These T cells migrate to B cell zones where they differentiate into follicular helper T cells (TFH) and move into the circulation to complete their maturation as helper T cells 2 (Th2). IL-4 secreting TFH cells in areas of B cells mediate IgE class switching in B lymphocytes, which attach to the surface of mast cells, basophils and eosinophils (not shown in figure), mediating the degranulation of these cells. IL-9 also stimulates mast cells. Th2 cells migrate to the airway epithelium and to the submucosa layer, where they secrete IL-5 and IL-13 to mediate the inflammatory response, with the accumulation of mast cells and eosinophils, airway remodeling and bronchial hyperreactivity.

After the challenge with the allergen, cytokines such as IL-33, derived from epithelial and inflammatory cells, have the ability to activate innate immune cells such as eosinophils, mast cells, basophils, innate lymphoid cells type 2 (ILC2), acting in combination with the stem cell factor (SCF) and the IgE receptor (Cherry et al., 2008; Schneider et al., 2009; Saenz et al., 2010; Silver et al., 2010; Ramadas et al., 2011). The activity of Th2 cytokines maintains the epithelial responses triggered by allergens, reacting to the cytokines IL-4 and IL-13, producing chemokines such as CCL11, CCL17 and IL-8, which, in turn, attract eosinophils, neutrophils, among other cells of the Th2 profile lingering asthma symptoms. This feedback loop of immune cell recruitment and inflammatory response contributes to the worsening of this disease (Matsukura et al., 2001; Lordan et al., 2002). Figure 2 represents the immune mechanism of allergic asthma. The complete cascade of inflammation and abnormal processes of attempts at repair generate structural changes in the walls of the airways of asthmatic individuals, contributing to tissue remodeling throughout the bronchial structure, which are more pronounced in the small airways (Saetta et al., 1991; Elias et al., 1999).

In asthmatic patients, the airway epithelium is fragile and, in some areas, absent in ciliated cells (Lackie et al., 1997). The maintenance of the integrity of the bronchial epithelial membrane depends on the presence of adherent junctions, which are responsible for binding and maintaining the cohesion of bronchial epithelial cells. Airway epithelial alterations involve desquamation, loss of hair cells and goblet cell hyperplasia (Aikawa et al., 1992; Carroll et al., 1993; Xiao Q et al., 2011). Due to the abnormal repair, epithelial desquamation leads to increased proliferation, contributing to thickening and fibrosis of the epithelium (Cohen et al., 2007), accompanied by an increase in the area of the subepithelial mucous glands and hyperplasia of Goblet cells (Ordoñez et al., 2001; Jenkins et al., 2003).

Another cytokine that plays an important role in the development of effects associated with bronchial remodeling is transforming growth factor β (TGF-β), produced by several cell types in the lungs, such as lymphocytes, fibroblasts, macrophages, eosinophils, and epithelial cells (Tagaya and Tamaoki, 2007). It has the ability to induce fibroblasts to express α-actin, which leads to their transformation into myofibroblasts (Batra et al., 2004). As part of abnormal repair, TGF-β induces goblet cell proliferation and the trans differentiation of ciliated cells and Clara cells into Goblet cells, increasing mucus production in the remodeling process (Le Cras et al., 2011). These mucous cells produce the mucin glycoprotein (MUC), the predominant form in the airways being MUC5AC; and the submucosal glands mainly produce MUC5B, with MUC5B being predominant under normal conditions and MUC5AC over-regulated in asthma (Evans et al., 1999; Kirkham et al., 2002; Rose and Voynow, 2006).

One of the most notable alterations associated with airway remodeling is the hypertrophy and hyperplasia of smooth muscle cell (Carroll et al., 1993). Additionally, mesenchymal cells undergo differentiation and migration to the smooth muscle cell layer, contributing to the overall increase. This increase in smooth muscle cell size and proliferation is a major contributor to the increased contractility seen in asthma, which is a primary underlying factor for bronchial hyperresponsiveness (Martin et al., 2000; Schmidt et al., 2003; Bergeron et al., 2009).

### 3.2 Asthma treatment

Current asthma pharmacotherapy is based on two fronts: bronchodilation or broncho protection, by direct inhibition of contraction or facilitation of relaxation of airway smooth muscle; and minimize inflammatory mediators that can induce bronchoconstriction and thus improve asthma symptoms (Pera and Penn, 2016). For the rapid control of asthma attacks and relief of symptoms, bronchodilators are used as first choice drugs, which act mainly by activating G protein-coupled receptors (GPCR)-G_s_ or antagonism of GPCR-G_q/11_. These agents are formed by β-adrenergic agonists, that activate GPCR-G_s_, such as salbutamol, salmeterol, and formoterol, and antagonists of muscarinic M_3_ receptors, such as ipratropium and tiotropium, or Cys-LT receptors, such as montelukast and zafirlukast, that inhibits GPCR-G_q/11_. Besides these, another class used in the treatment of asthma, but which does not act directly on the GPCRs, are phosphodiesterase inhibitors, enzymes that degrade cyclic nucleotides, the example of theophylline (Prado et al., 2014).

The airway inflammation is controlled through the use of steroidal anti-inflammatory drugs, corticosteroids, orally (OCS) or inhaled (ICS), which act in the long term by inhibiting the inflammatory process, such as fluticasone and budesonide (Pera and Penn, 2016). Since their emergence and introduction in the treatment of asthma, corticosteroids have presented problems related, especially, to their side effects, so that new drugs in the class have been and are being developed with the aim of improving their potency and, mainly, their systemic effects (Colice, 2006). Common side effects of corticosteroids include oral candidiasis, glaucoma, cataracts, skin bruising and osteoporosis. However, it is important to note that the likelihood of experiencing these complications is significantly lower with inhaled versus oral glucocorticoids (Gina, 2022). Other anti-inflammatory drugs are cromoglycates that act by stabilizing mast cells, such as cromolyn sodium, preventing mast cell degranulation. Both corticosteroids and cromolyn are used to treat mild persistent asthma (Lane and Lee, 1996).

New classes of drugs have been developed for treating severe asthma, including anti-IgE antibodies such as omalizumab, especially for corticosteroid-resistant patients (Humbert et al., 2014). Furthermore, new drugs studied include anti-IL-13 antibodies, with promising results in clinical studies (Hodsman et al., 2013; De Boever et al., 2014; Powell et al., 2015). So far, no therapy for asthma has been used to prevent the airway remodeling. As the first-line choice, anti-inflammatory drugs continue to be used, being crucial in the management of the disease. Although the emergence of new pharmacological therapies for asthma has been strengthened, there is still a need for the development of additional effective treatments (Berair and Brightling, 2014), in this sense, knowledge of the ion channels involved in the pathophysiology of asthma is of fundamental importance for a possible more effective therapeutic strategy.

## 4 Ion channels involved in asthma

### 4.1 Transient receptor potential (TRP)

#### 4.1.1 Molecular structure

TRP cationic channel superfamily, composed of 28 members, is divided into seven subfamilies in mammalians. Of these, six are found in humans: ankyrin (TRPA, 1 member), canonical (TRPC, 6 members), melastatin (TRPM, 8 members), mucolipin (TRPML, 3 members), polycystine (TRPP, 3 members), and vanilloid (TRPV, 6 members) (Zhao et al., 2021).

Structurally, TRP channels share a common feature, consisting of six transmembrane domains (S1-S6) and a pore-forming loop between the fifth and sixth segments (Benemei et al., 2015). These channels have four pore-forming TRP protein subunits, and can be expressed as homo- and heterotetramers (Moran et al., 2011). Both the intracellular C- and N-terminal regions regulate channel assembly and function through the present domains. In addition to these structures shared by all TRPs, others are also present among some subfamilies. TRPM, TRPA and TRPC channels have a coiled domain in the C-terminal region that stabilizes the tetrameric assembly. TRPA and TRPV channels have ankyrin repeats in the N-terminal region that are capable of transducing conformational changes to the pore. Thus, one of the structural characteristics that differentiate the subfamilies of this channel are the coiled-coil regions, TRP motifs and the number of ankyrin repeats (Hilton et al., 2019).

#### 4.1.2 Molecular physiological and biophysical properties

TRP channels function as detectors that react to alterations in the cellular surroundings, encompassing variations in temperature, mechanical stretch, oxidative processes, osmolarity, and pH levels. They are also stimulated by the activation of GPCR and the action of several intracellular molecules, including phospholipase C (PLC), phosphatidyl inositol 4,5-bisphosphate (PIP_2_), diacylglycerol (DAG), protein kinase A (PKA) and C (PKC), adenosine triphosphate (ATP), calmodulin and arachidonic acid metabolites, in addition to agonists such as capsaicin, phorbol esters, menthol, eucalyptol, mustard oil and cinnamaldehyde (Castillo et al., 2018; Michalick and Kuebler, 2020).

Most TRP channels, with the exception of TRPM4 and TRPM5, exhibit permeability to both Ca^2+^ and monovalent cations. However, the channels permeable to Ca^2+^ display limited selectivity for this ion, as their conductance rate in relation to Na^+^ (P_Ca_/P_Na_) typically ranges from 0.3 to 10. Notwithstanding, TRPV5 and TRPV6 stand out as selective channels for Ca^2+^ with P_Ca_/P_Na_ > 100. TRP activation, therefore, also serves as an important Ca^2+^ influx pathway, contributing to Ca^2+^ signaling (Guibert et al., 2011; Hasan and Zhang, 2018).

Due to their wide expression and distribution, TRP channels present significant roles in physiological processes. These processes include crucial sensory functions like pheromone signaling, nociception and thermoregulation (Wang and Siemens, 2015; Laing and Dhaka, 2016); homeostatic functions such as reabsorption of Ca^2+^ and Mg^2+^, regulation of osmolarity, metabolism and adipogenesis (Uchida et al., 2017; Dhakal and Lee, 2019; Startek et al., 2019); besides to muscle contraction and vasomotor control (Lonso-Carbajo et al., 2017).

#### 4.1.3 Expression profile and contributions to asthma

**FIGURE 3:**
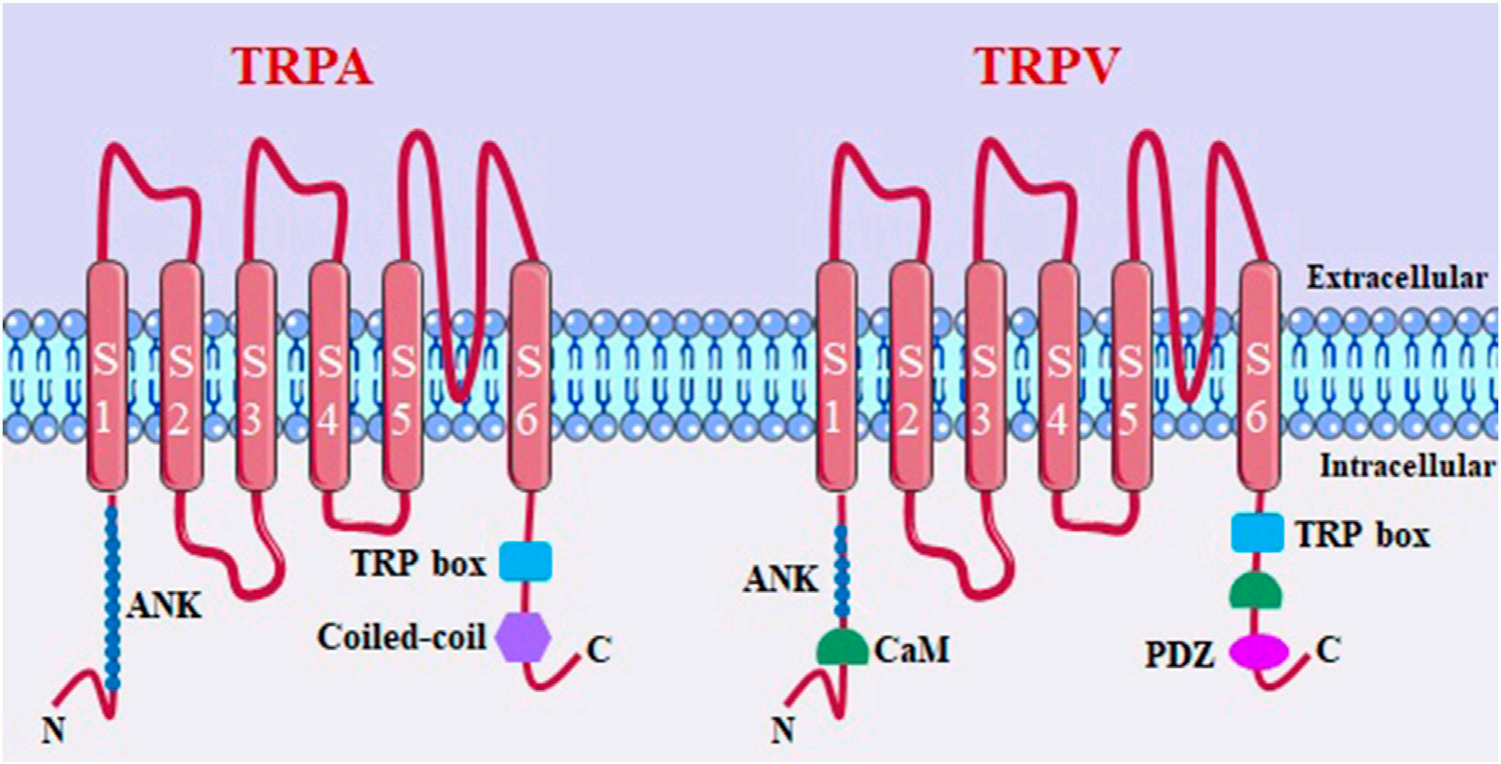
TRPA and TRPV channel structure. ANK, ankyrin repeats (varies in number among members of subfamilies); coiled-coil domains; TRP box; CaM, calmodulin binding site; PDZ specific reason for binding to PDZ.

Within the respiratory system, in different cell types, several TRP channels are present and expressed at varying levels. However, among them, TRPA1, TRPV1, and TRPV4 are particularly noteworthy due to their strategic localization, greater quantity, and role in maintaining airway function (Figure 3) (Logu et al., 2016). The expression of TRPA1 has been observed in sensory neurons that innervate the airways, immune cells implicated in the inflammatory response associated with chronic obstructive pulmonary disease (COPD) and asthma. These immune cells include mast cells, B cells, and CD4^+^ and CD8^+^ T cells (Banner et al., 2011).

Additionally, TRPA1 was evidenced to be present in human lung bronchial epithelial cell lines (Mukhopadhyay et al., 2011; Büch et al., 2013; Wortley et al., 2016), and it is present in sensory neurons, bronchial epithelial cells, lung dendritic and smooth muscle cells, and CD4^+^ T cells (Bertin et al., 2014; Samivel et al., 2016). TRPV4, on the other hand, can be found in airway smooth muscle, vascular endothelial cells, macrophages, alveolar, bronchial, and tracheal epithelium (Rosenbaum et al., 2020).

In recent years, there has been evidence highlighting the significant role of TRP channels in respiratory diseases, including asthma (Kytikova et al., 2020). The activation of these channels contributes to inflammatory processes and structural alterations in the respiratory tract, leading to bronchoconstriction (Taylor-Clark, 2016). Since activators of TRP channels, such as reactive oxygen species or cigarette smoke, can exacerbate asthma symptoms, the involvement of these channels in the pathophysiology of this disease becomes evident (Preti et al., 2012).

TRPA1 appears to have involvement in multiple aspects of asthma, as the activation of this channel leads to increase of airway hyperreactivity, a characteristic feature of this disease (Tränkner et al., 2014). Additionally, studies have shown a correlation between variants of the TRPA1 gene and childhood asthma, further suggesting the participation of this channel in the pathogenesis of asthma. Other studies also showed that knockout mice for this channel (TRPA1^−/−^) showed reduced infiltration of immune cells into the bronchoalveolar fluid in the ovalbumin-induced asthma model, highlighting the contribution of this channel to airway inflammation in mice model asthma (Gallo et al., 2017). Moreover, TRPA1 expressed in sensory neurons that innervate the airways can be activated by inhaled irritants, resulting in influx of calcium ions and release of inflammatory substances. These substances may influence the recruitment of neutrophils and eosinophils in the lungs, contributing to the inflammatory response associated with respiratory conditions such as asthma (Reese et al., 2020).

Of these released substances, it can be mentioned pro-inflammatory neuropeptides (neurokinin A, substance P and the peptides related to the calcitonin gene) responsible for the development of neurogenic inflammation in the airways. These, in turn, mediate the initial inflammatory response, such as vasodilation, leukocyte extravasation, mucus hypersecretion, and airway constriction (Yang and Li, 2016). This response was also observed through the positive modulation of TRPV1 channels (Moran et al., 2011; McMahon et al., 2015), taking into account that the neurogenic inflammation caused by the release of these neuropeptides contributed to the worsening of asthma. This condition also occurred due to the production of cytokines such as tumor necrosis factor-α, IL-1, IL-6 and IL-8 by human bronchial epithelial cells, considering that these factors can cause bronchoconstriction (Lee and Yang, 2012; Song et al., 2017; Kytikova et al., 2020).

Human studies reveal that patients with severe asthma have been reported to exhibit an increase in TRPV1 expression in the airway epithelium (McGarvey et al., 2014). Other data showed that compared to healthy children, asthmatic children presented an increase in TRPV1 gene expression (Ren et al., 2015). In addition, exposure to inflammatory stimuli, such as capsaicin and high temperatures, has been reported to increase TRPV1 expression (Sadofsky et al., 2012). Moreover, it has been observed that the loss of this channel-specific gene is related to reduced susceptibility to active childhood asthma. This suggests that variations in TRPV1 function may play a role in modulating asthma risk and severity in affected individuals. This suggests that variations in TRPV1 function may play a role in modulating the risk and severity of asthma in affected individuals (Cantero-Recasens et al., 2010).

Baker et al. (2016) showed that in TRPV1 knockout mice (TRPV4^−/−^) in a house dust mite-induced asthma model, a reduction in IgE levels, inflammation and airway hyperresponsiveness was observed. In addition, they showed the role of a blocker of this channel, XEN-D0501, and although IgE levels were not altered, the action of this inhibitor resulted in a reduction in the airway inflammation profile, suggesting that TRPV1 blockade attenuates the allergic asthma phenotype.

Studies have provided evidence that TRPV4 plays a regulatory role in allergic asthma. In an mice model of allergic asthma, it has been observed that TRPV4 can modulate airway wall thickness, collagen synthesis, recruitment of goblet cells, expression of TGF-β and fibrosis remodeling in the airways (Scheraga et al., 2017). Additionally, another study using a mouse model of allergic asthma induced by ovalbumin found that exposure to high relative humidity and formaldehyde intensified the activation of TRPV4 in the lungs. This, in turn, resulted in the inflammation and mucus hypersecretion in the airways, leading to asthma exacerbation (Duan et al., 2020). In a mite-induced asthma model, knockout mice for this channel (TRPV4^−/−^) was protected from airway remodeling. Likewise, isolated lung fibroblasts from asthmatic individuals showed increased TRPV4 activity (Gombedza et al., 2017).

Therefore, based on these studies, it is suggested that reduced activation of TRP family channels, especially TRPA1, TRPV1 and TRPV4, may be potential targets for new therapies for asthma (Dietrich, 2019; Reese et al., 2020).

### 4.2 Storage-operated Ca^2+^ channels (SOCs)

#### 4.2.1 Molecular structure

Ca^2+^ release-activated Ca^2+^ channels (CRAC) are widely distributed category of store-operated Ca^2+^ channels (SOCs). These channels are triggered due to the depletion of Ca^2+^ within the endoplasmic reticulum (ER) lumen. The function of CRACs depends on two classes of membrane proteins: the Ca^2+^-sensing stromal interacting molecules (STIM1 and STIM2), located in the ER membrane; and the plasma membrane channel pore-forming proteins (Orai1, Orai2 and Orai3). All types of STIM and Orai proteins are present in mammals, however, STIM1 and Orai1 are the most studied (Niu et al., 2020; Zhang et al., 2020).

**FIGURE 4:**
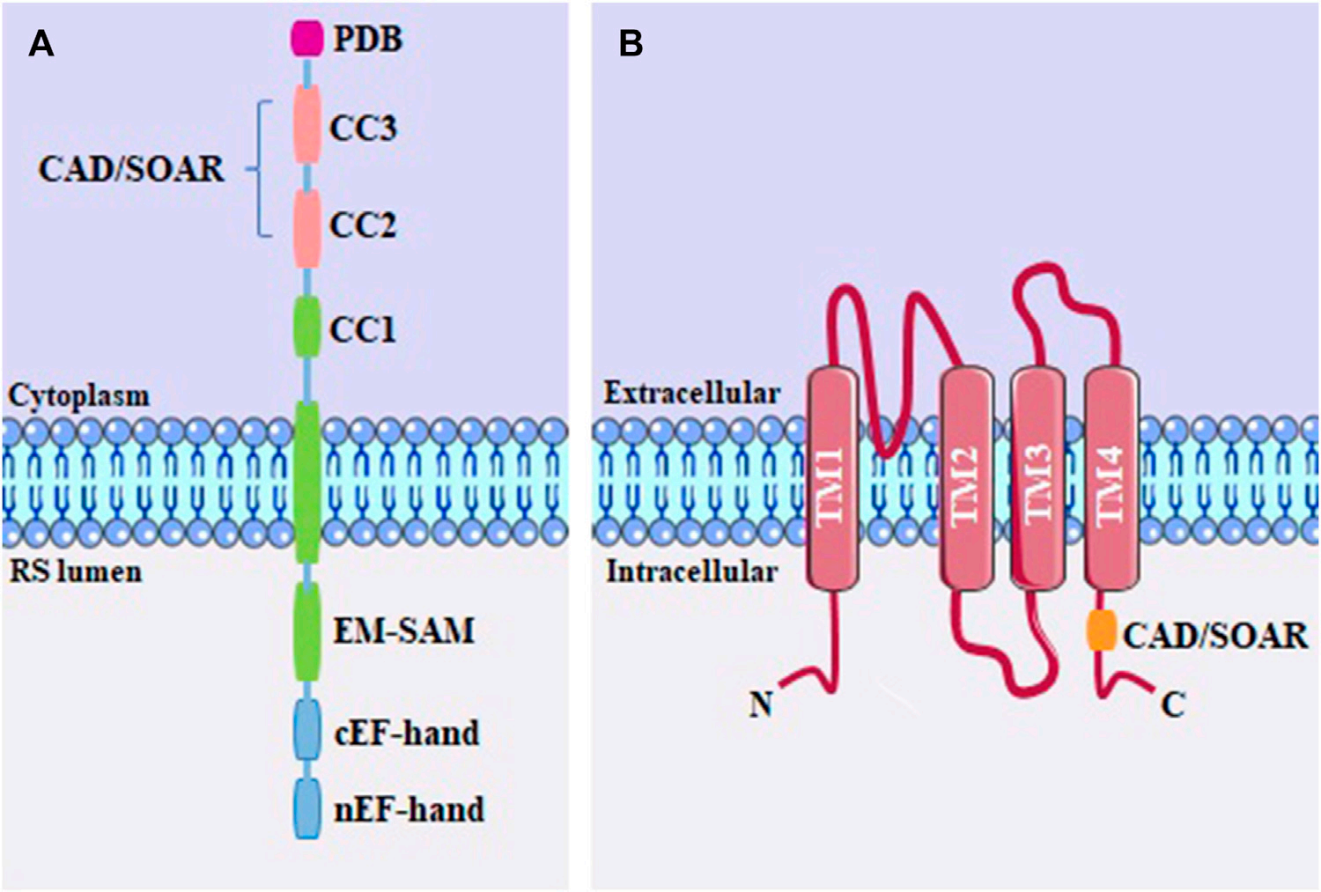
**(A)** STIM monomeric structure, including the PDB domain; the coiled-coil domains (CC1, CC2 and CC3); CAD/SOAR domain; sterile alpha motif (SAM) and EF-hand; **(B)** Structure of the Orai channel, containing four transmembrane domains (TM1-TM4) and the CAD/SOAR binding domain.

The STIM protein has in its structure (Figure 4A) facing the ER lumen, EF-hand domains (a helix–loop–helix structural domain or motif found), a canonical that binds to Ca^2+^, and a non-canonical structure, in addition to a sterile alpha motif (EF-SAM). The cytoplasmic region has a putative coiled-coil (CC1) domain; an STIM-Orai activation domain, called CAD/SOAR, which also comprises the coiled-coil CC2 and CC3; as well as a polybasic domain (PDB), which interacts with molecules in the plasma membrane, including PIP_2_ (Bakowski et al., 2020; Lewis, 2020). Considered dimeric at rest, STIM1 is stabilized by hydrophobic interactions and hydrogen bonds between CAD/SOAR monomers. To avoid spontaneous activation of STIM1, the EF-SAM luminal regions are kept separate when bound to Ca^2+^, allowing the CC1 cytosolic domain to interact with CAD/SOAR, keeping it close to the ER membrane (Korzeniowski et al., 2017).

The Orai1 channel (Figure 4B) was revealed to be a hexameric oligomer, where each subunit is a protein spanning four transmembrane expansion domains (TM1-4) (Hou et al., 2012). The pore of the Orai1 channel is formed by the N-terminal transmembrane helix (TM1). The second and third transmembrane helix (TM2 and TM3) are packed around the pore in each subunit (Cai et al., 2016). The C-terminal region of the protein contains the fourth transmembrane helix (TM4) with an extended cytoplasmic segment. This cytoplasmic extension is recognized as the primary site for interaction with STIM proteins (Nwokonko et al., 2017; Zhou et al., 2017).

#### 4.2.2 Molecular physiological and biophysical properties

When the concentration of Ca^2+^ in the ER decreases, Ca^2+^ dissociates from the EF-hand luminal domain. This dissociation leads to the unfolding of the EF-SAM domain, resulting in a conformational change in the cytosolic region, that promotes the formation of STIM1 oligomers and facilitates the dissociation of the CC1 domain from CAD/SOAR. After Ca^2+^ depletion, the STIM1 oligomers migrate to the cortical ER associate with the plasma membrane, at a distance short enough for the direct interaction between STIM1 and Orai1 through the exposure of the CAD/SOAR domains, which in turn interact with Orai1 to activate this channel, resulting in influx of Ca^2+^ (Bakowski et al., 2020; Baraniak et al., 2020; Fahrner et al., 2020).

Activation of CRAC channels occurs proportionally to the reduction in Ca^2+^ levels, allowing for the entry of extracellular Ca^2+^ to replenish the depleted stores (Qiu and Lewis, 2019). These channels play important roles in regulating various cellular functions, including cell proliferation, gene expression, cell migration, and apoptosis (Kappel et al., 2019). SOC signaling is particularly essential for several aspects of T cell function, such as T cell development and differentiation, stimulation of CD8^+^ T cells followed by release of cytotoxic granules, cytokine production, and control of mast cell and neutrophil function (Vaeth and Feske, 2018; Vaeth et al., 2020).

Under physiological conditions, ER Ca^2+^ stores are depleted by the action of second messenger inositol triphosphate (IP_3_). IP_3_ is generated from the hydrolysis of the membrane phospholipid, PIP_2_, by the action of PLC enzymes. Several types of PLC are stimulated by different G proteins or receptor tyrosine kinases, depending on the tissue and the ligand (Lopez et al., 2020). IP_3_ can then bind to its receptors (IP_3_R) on the ER membrane, favoring the Ca^2+^ release to the cytoplasm. In excitable cells, ryanodine receptors (RyR) are reported to play a greater role in luminal ER Ca^2+^ depletion than IP_3_R. That said, after the decrease of Ca^2+^ stored in the ER, CRACs are activated, providing a sustained influx of Ca^2+^ (Zhu et al., 2017).

Interestingly, the stoichiometry of the STIM-Orai coupling is variable (Yen and Lewis, 2019). The optimal activation of the Orai channel occurs with a coupling ratio of 2:1, suggesting that a single hexameric Orai1 channel interact with six STIM1 dimers (Hoover and Lewi, 2011). However, structural studies present an alternative perspective, proposing a bimolecular interaction model. According to this model, two adjacent Orai1 subunits interact with an STIM1 dimer (Maus et al., 2015).

#### 4.2.3 Expression profile and contributions to asthma

CRAC channels represent potential targets for the treatment of respiratory disorders, since they are involved in several diseases such as those related to the immune system. In airway immune cells, CRAC inhibition modulates the chronic inflammation seen in asthma. Furthermore, CRAC channels are expressed in airway smooth muscle and epithelial cells, making them attractive targets for therapeutic interventions in asthma (Johnson and Trebak, 2019; Bakowski et al., 2020). Studies have shown that the expression of STIM1 and Orai1 proteins was positively modulated in smooth muscle cells of tracheal and bronchial tissue isolated from ovalbumin-challenged mice with asthma. The reduced gene expression of these proteins significantly inhibited chemotactic migration and smooth muscle cell proliferation, highlighting the role of STIM1 and Orai1 as targets for smooth muscle remodeling during asthma (Spinelli et al., 2012).

Previously, experimental studies have also shown the significant involvement of SOC in a model of allergic asthma in guinea pigs. These results showed decreased airway hyperreactivity at single doses of a STIM-Orai coupling blocker, 3-fluoropyridine-4-carboxylic acid (FPCA), as well as decreased basal airway reactivity and presented anti-inflammatory response in long-term administration of this blocker (Šutovská et al., 2013).

Šutovská et al. (2015) reported the effect of long-term administration of FPCA blocker on respiratory epithelial cells in conditions of allergic asthma induced by ovalbumin in guinea pigs. The reduction in cytokine levels confirmed an anti-inflammatory effect of this blocker (Šutovská et al., 2015). Furthermore, according to Šutovská et al. (2021), the effects of the STIM-Orai antagonist SKF 96365 were investigated through inhalation in guinea pigs with ovalbumin-induced airway remodeling. Three increasing doses of the antagonist were administered, and its impact on hyperresponsiveness was evaluated. Histology and immunohistochemical analyzes confirmed the prevention of airway remodeling alterations induced by repetitive exposure to ovalbumin in inhibition of the STIM-Orai pathway. The researchers observed that the inhibition of tissue remodeling promoted by SKF 96365 occurred through suppression of IL-4, IL-5, IL-12, IL-13, interferon γ (INF-γ) and TNF-ɑ pathway.

The inhibition or reversal of airway tissue remodeling in asthma is a therapeutically important target. Thus, the STIM-ORAI pathway stands out as a possible mechanism responsible for this effect, making store-operated Ca^2+^ channels as promising therapeutic targets for an effective treatment for asthma (Johnson and Trebak, 2019; Šutovská et al., 2021).

### 4.3 Intermediate and big conductance Ca^2+^-activated K^+^ channels (IK_Ca_ and BK_Ca_)

#### 4.3.1 Molecular structure

Ca^2+^-activated K^+^ channels (K_Ca_) comprise several ion channels with different characteristics. Based on their conductance levels, Ca^2+^-activated K^+^ channels can be categorized into three subfamilies: small conductance (SK_Ca_), intermediate conductance (IK_Ca_), and large conductance (BK_Ca_). Among these subfamilies, IK_Ca_ and BK_Ca_ channels are particularly involved in the pathogenesis of asthma. These channels play significant roles in regulating airway smooth muscle tone and bronchial hyperresponsiveness, key factors contributing to asthma symptoms and airway constriction (Kshatri et al., 2018).

**FIGURE 5:**
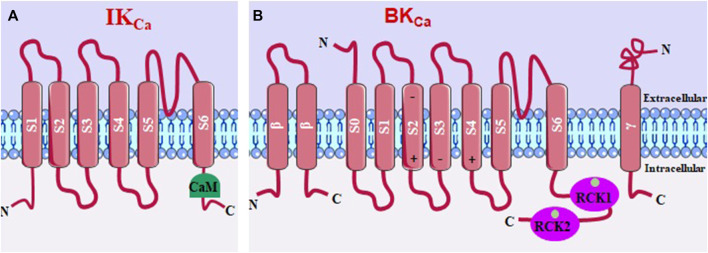
**(A)** IK_Ca_ structure, with 6 transmembrane segments and the calmodulin binding domain (CaM). **(B)** BK_Ca_, with seven transmembrane segments and two Ca^2+^-sensitive domains that regulate K^+^ conductance.

IK_Ca_ (or K_Ca_3.1) channels have intracellular C- and N-terminal regions and four α subunits, each with six transmembrane helices (S1-S6). The pore-forming region comprises the S5 and S6 helices, being K^+^ selective (Figure 5A) (Adelman, 2016; Kshatri et al., 2018). BK_Ca_ (or K_Ca_1.1) channels have a multimeric structure, comprising four identical pore-forming α subunits and up to four β and γ regulatory subunits (Figure 5B). These channels have seven transmembrane segments (S0-S6), with S0 being a domain that leads the N-terminal portion to the extracellular side. Furthermore, within segments S2, S3, and S4 are charged amino acid residues that play roles in voltage detection (Al-Karagholi et al., 2020).

#### 4.3.2 Molecular physiological and biophysical properties

IK_Ca_ channels are independent of membrane voltage and are activated by a small increase in [Ca^2+^]_c_ through a mechanism that consists of binding this ion to calmodulin (CaM), forming a complex that activates the calmodulin binding domain (CaMBD) in the C-terminal region of the IK_Ca_ (Adelman, 2016; Kshatri et al., 2018).

The β1 and β4 subunits stabilize the voltage detection domain of BK_Ca_ channels in an active conformation, leading to increased channel activity and promoting membrane potential hyperpolarization. This results in more negative values of the membrane potential. In contrast, the β2 and β3 subunits favor an inactive conformation of BK_Ca_ channels, limiting K^+^ efflux and membrane hyperpolarization (Hite et al., 2017). The cytosolic C-terminal region of BK_Ca_ channels is relatively large and consists of two K^+^ conductance regulator domains (RCK1 and RCK2) that contain a high-affinity Ca^2+^ binding site. When Ca^2+^ binds to these sites, it directly activates BK_Ca_ channels without involving the calcium-binding protein calmodulin (CaM). This direct interaction with Ca^2+^ leads to the opening of the channel pore and the efflux of K^+^ ions (Lee and Cui, 2010; Yuan et al., 2012; Hoshi et al., 2013).

#### 4.3.3 Expression profile and contributions to asthma

IK_Ca_ channel is expressed in mast cells, macrophages, fibroblasts, T lymphocytes, epithelial cells and smooth muscle cells in the airways of normal and asthmatic individuals (Girodet et al., 2013; Pelletier and Savignac, 2018), and can control several cellular processes, such as proliferation, chemotaxis, cell activation and smooth muscle responsiveness (Van der Velden et al., 2013). In addition, IK_Ca_ participates in the pathophysiology of rheumatoid arthritis, multiple sclerosis, atherosclerosis, fibrosis, rhinitis, and asthma (Ohya and Kito, 2018).

In humans, BK_Ca_ channels have a wide expression profile, being found in various tissues, including respiratory systems (Latorre et al., 2017; Gonzalez-Perez and Lingle, 2019). The opening of BK_Ca_ regulates several physiological processes, such as smooth muscle contraction, hormonal secretion, neuronal excitation and gene expression (Yang et al., 2015). Studies have reported that mutations in the KCNMA1 gene encoding the BKCa channel, known as KCNMA1-linked channelopathy in humans, are associated with neurological conditions such as movement disorders, seizures and developmental delay. In addition, there is evidence of the association of other polymorphisms in this channel with diseases such as asthma (Bailey et al., 2019).

Yu et al. (2013) demonstrated the role of IK_Ca_ in the pathogenesis of inflammation and airway remodeling in ovalbumin-induced allergic asthma in mice. Thus, it was reported that in this disease model, IK_Ca_ expression was elevated, especially in bronchial smooth muscle cells. In addition, blocking this channel by triarylmethane (TRAM-34) resulted in the inhibition of inflammation, remodeling and airway hyperresponsiveness, as well as in the interruption of bronchial smooth muscle cell proliferation in asthmatic humans (Yu et al., 2013).

In a mouse model of chronic asthma, studies have demonstrated that blocking IK_Ca_ channels using TRAM-34 has beneficial effects. Treatment with TRAM-34 has been shown to reduce airway remodeling, decrease airway eosinophilia and collagen deposition. Additionally, when TRAM-34 was used in combination with preventive and therapeutic treatments, it was found to reduce bronchial hyperresponsiveness to methacholine. These findings suggest that targeting IK_Ca_ channels with blockers like TRAM-34 may hold potential as a therapeutic strategy for managing asthma and its associated symptoms (Girodet et al., 2013). Another study has shown that the administration of senicapoc, an IK_Ca_ blocker, was effective in reducing bronchoconstriction, pulmonary resistance, airway hyperresponsiveness to carbachol and eosinophilia in bronchoalveolar fluid in an ovine model of mite-induced experimental asthma (Van der Velden et al., 2013).

Inhibition of inflammation, remodeling, and airway hyperresponsiveness were also observed through deletion of the IK_Ca_ gene. In this study, it was also shown, for the first time, that the suppression of the proliferation of bronchial smooth muscle cells in asthmatic humans occurred because of the inhibition of calcium influx via TRPV4 due to the deletion of the IK_Ca_ gene, given that these channels are colocalized and functionally interact to regulate [Ca^2+^]_c_ in these cells (Yu et al., 2017). This is because in airway smooth muscle cells, the IK_Ca_ channels act as Ca^2+^ detectors and amplifiers, since the membrane hyperpolarization caused by the activation of this channel positively regulates Ca^2+^ influx (Guéguinou et al., 2014).

BK_Ca_ polymorphisms have been associated with susceptibility to asthma in an African population (Mantese et al., 2011). Furthermore, a BK_Ca_ activator, NS1619, decreased *in vivo* and *in vitro* airway hyperreactivity, ciliary beat frequency, inflammatory cell infiltration, pro-inflammatory cytokines and exhaled nitric oxide in a guinea pig model of ovalbumin-induced allergic asthma (Kocmalova et al., 2015).

Studies have also shown that rottlerin, a BK_Ca_ activator, attenuated both airway hyperreactivity *in vivo* and *in vitro*, and infiltration of inflammatory cells in the lung, by reducing the production of T_H_2 profile cytokines in ovalbumin and mite-induced asthma models (Goldklang et al., 2013). In addition, andolast, another BK_Ca_ activator, is in clinical phase III for a potential treatment of allergic rhinitis, COPD, and bronchial asthma (Al-Karagholi et al., 2020).

Based on the evidence implicating IK_Ca_ and BK_Ca_ channels in asthma, there is growing support for considering these channels as promising targets for the treatment of the disease. The involvement of these channels in regulating airway smooth muscle tone, bronchial hyperresponsiveness, and inflammation highlights their potential as therapeutic targets. By modulating the activity of IK_Ca_ and BK_Ca_ channels, it may be possible to mitigate the pathological features of asthma and improve symptoms. Further research and development of specific modulators or blockers for these channels could lead to novel therapeutic interventions for the treatment of asthma.

### 4.4 Calcium-activated chloride channel (TMEM16A)

#### 4.4.1 Molecular structure

Ca^2+−^activated chloride channels (CaCCs) are channels that allow the passage of chloride ions (Cl^−^) and can be activated by the presence of intracellular calcium and also by voltage. A specific type of CaCCs, called anoctamine-1 (ANO1) or transmembrane protein 16A (TMEM16A), was initially described in *Xenopus* oocytes. The ANO1/TMEM16A gene encodes a protein that belongs to a family known as anoctamins, which is composed of 10 members (ANO1–ANO10) in mammals (Schroeder et al., 2008; Yang et al., 2008; Kamaleddin, 2018).

**FIGURE 6:**
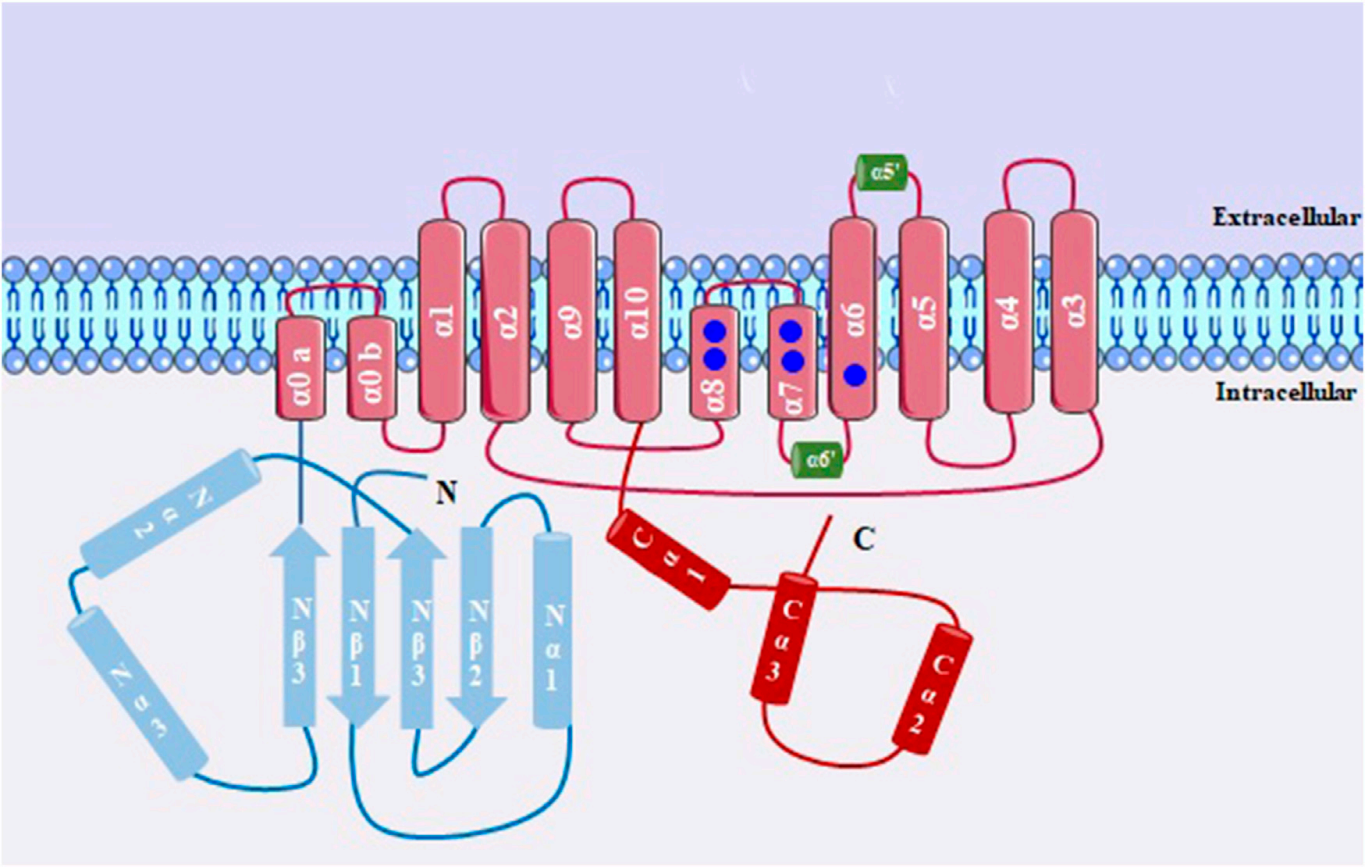
Structure of a subunit of TMEM16A, with 10 transmembrane domains. Between α3-α7 there is the formation of the channel pore; Ca^2+^ binding sites is shown in blue circles.

TMEM16A channels (Figure 6) are formed by transmembrane homodimers, in which each subunit is regulated independently (Jeng et al., 2016). They have N- and C-terminal domains and the transmembrane domain starts with two short α-helices (α0a and α0b), followed by ten membrane-spanning segments (α1–α10). Between helices, there are calcium-binding regions, and the intracellular loop between α6–α8 contains six amino acids (N650, E654, E702, E705, E734 and D738) that participate in the formation of principal calcium-binding pocket, thus regulating the opening of this channel. The α3–α7 helices form the pore of the channel (Brunner et al., 2014; Paulino et al., 2017).

#### 4.4.2 Molecular physiological and biophysical properties

Most naturally expressed CaCCs are sensitive to low [Ca^2+^]_c_ (100 nM to 1–2 μM), so that a considerable increase in [Ca^2+^]_c_ leads to inactivation of this channel (Schreiber et al., 2010; Yang et al., 2008). Associated with this, changing the membrane potential to fewer negative values can also cause activation of these channels (Xiao C et al., 2011). This occurs because the calcium binding site is located in the transmembrane region of the channel, which is also sensitive to changes in membrane potential. Furthermore, the sensitivity of the channel to activation by cytosolic calcium decreases when the membrane potential is hyperpolarized (Yang et al., 2008; Pedemonte and Galietta, 2014).

When activated, the chloride ion conductance in a single channel is 0.5–5 pS. This channel also showed relative permeability for the following monovalent anions, in increasing order of permeability: NO_3_^−^, I^−^, Br^−^, Cl^−^ and F^−^ (Yang et al., 2008; Alexander et al., 2021). Studies have also shown that TMEM16A can be activated in the presence of other divalent ions such as Ba^2+^, Sr^2+^ and Ni^2+^ (Yuan et al., 2013). Previous research has established that activation of phospholipase C leads to the hydrolysis of PIP_2_, producing IP_3_, which in turn releases calcium from intracellular compartments, potentially activating TMEM16A (Cruz-Rangel et al., 2015; Jesús-Pérez et al., 2018).

#### 4.4.3 Expression profile and contributions to asthma

These channels are found in a variety of organisms and tissues, suggesting their functional diversity. TMEM16A has already been reported to be present in neuronal, vascular, myocardial, epithelial and smooth muscle cells (Large and Wang, 1996; Pedemonte and Galietta, 2014; Rottgen et al., 2018). In humans, TMEM16A is widely expressed in the respiratory tract, from the bronchi to the alveoli (Kunzelmann et al., 2012). In addition, it is largely related to the regulation of transepithelial transport and smooth muscle contraction (Pedemonte and Galietta, 2014). TMEM16A hyperactivity plays a role in neuropathic pain, cell migration and proliferation in different types of cancer, as well as in asthma (Zhang et al., 2013; Crottès and Jan, 2019; Kulkarni et al., 2023).

Studies suggest that the increase in TMEM16A activity is related to the pathogenesis of asthma, so that the inhibition of this protein characterizes a therapeutic alternative for this disease, especially related to the control of mucus hypersecretion, a potentially serious characteristic that culminates in airway obstruction. Thus, regulation of the expression of secretory cells and mucus secretion are shown to be important targets (Liu et al., 2021).

The upregulation of TMEM16A in airway smooth muscle cells was also confirmed by Zhang et al. (2013). It has been highlighted that activation of TMEM16A contributes to agonist-induced contraction, which activate these chloride channels to depolarize the membrane. Thus, deletion of TMEM16A rendered Ca^2+^ sparks incapable of activating chloride efflux and weakened caffeine and methacholine-induced muscle fiber shortening in knockout TMEM16A^−/−^ airway smooth muscle cells. Furthermore, TMEM16A blockers niflumic acid and benzbromarone, prevented hyperresponsiveness *in vivo* and *in vitro* in an ovalbumin-induced asthma model in C57BL/6 mice (Zhang et al., 2013).

Increased expression of TMEM16A on the surface of airway epithelial secretory cells has been reported both from C57BL/6 mice sensitized and challenged with ovalbumin and from asthmatic patients. They also showed that the inhibition of this protein by blockers such as benzbromarone and dichlorophen negatively modulated mucus secretion in human epithelial cells, in addition to reducing contraction in response to methacholine in smooth muscle cells of the airways of mice and humans (Huang et al., 2012).

Kondo et al. (2017) demonstrated overexpression of TMEM16A in the apical portion of the epithelium with goblet cell metaplasia in the trachea of guinea pigs sensitized with ovalbumin, associated with an increase in chloride ion conductance through this protein. Furthermore, it was shown that the presence of the blockers T16Ainh-A01, benzbromarone and niflumic acid in the culture of tracheal epithelial cells, the increase in chloride ion transport induced by the calcium-dependent agonist UTP was inhibited. T16Ainh-A01 also inhibited the mucus secretion that was shown in the trachea of sensitized animals, suggesting that TMEM16A inhibitors may be viable in inhibiting hypersecretion in asthma (Kondo et al., 2017).

Based on these data, there are indications that TMEM16A may be a unique therapeutic target for the treatment of asthma. TMEM16A-CaCCs channel blockers have the potential to treat both mucus hypersecretion and airway hyperresponsiveness in animal models, and may positively impact asthma symptoms.

### 4.5 Cystic fibrosis transmembrane conductance regulator (CFTR)

#### 4.5.1 Molecular structure

CFTR belongs to ATP-binding cassette (ABC) superfamily, a family with 48 members, grouped into 7 subfamilies (ABCA-G). CFTR belongs to ABCC subfamily, subtype 7 being the only one that functions as an ion channel, transporting mostly chloride ions, but also bicarbonate and other ions. ABC transporters use the chemical energy of ATP hydrolysis to transport substrates against the electrochemical gradient, with the exception of CFTR that conduct anions in favor of the electrochemical gradient (Dean and Annilo, 2005; Gadsby et al., 2006).

**FIGURE 7:**
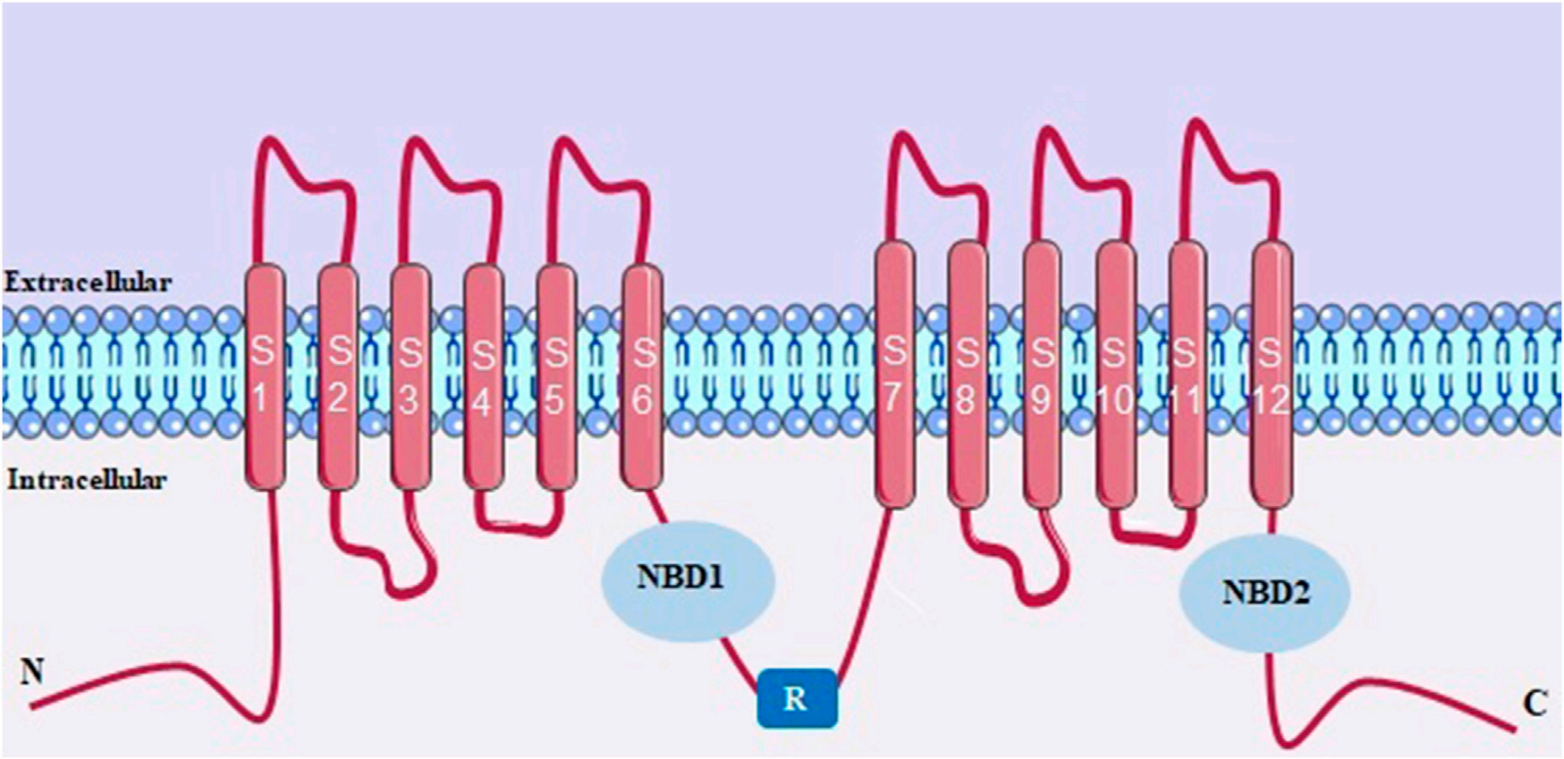
CFTR structure with two canonical transmembrane domains (TMDs). NBD, cytosolic nucleotide-binding domain. R, regulatory domain.

The CFTR protein is structurally (Figure 7) characterized by having two canonical transmembrane domains (TMDs) and each domain has 6 transmembrane segments, being S1-S6 in TMD1 and S7-S12 in TMD2, these being the structures responsible for the formation of the channel pore. Each TMD is followed by a cytosolic nucleotide-binding domain (NBD1 and NBD2). This channel also has a unique regulatory domain (R), located between TMD-NBD complexes that contain multiple sites for phosphorylation (Sohma and Hwang, 2015).

#### 4.5.2 Molecular physiological and biophysical properties

The literature describes that CFTR channel can present in 2 conformations, the open one, requires phosphorylation of R domain by protein kinase A (PKA) and the ATP binding site induces the opening of the channel and passage of ions. This channel has 2 distinct sites for ATP, “consensus” which is catalytically active and responsible for ATP hydrolysis and “degenerate” which has no catalytic activity. The ATP hydrolysis that occurs in the “consensus” induces pore closure. Pore opening in absence of ATP or its closure without ATP hydrolysis may also occur, but this is rare (Aleksandrov et al., 2002; Csanady et al., 2010; Wang et al., 2010).

The other conformation is the closed one, which occurs after phosphorylation of the R domain, inducing the folding of nucleotide-binding domains that interact with the transmembrane domains, leading to ATP hydrolysis and altering the channel conformation from an open state to closed one. In this conformation, the NBDs are separated by approximately 20 Å (Liu et al., 2017).

When analyzing the CFTR conductance in isolation, a concentration-dependent saturation of chloride was observed, suggesting a general affinity for the pore of approximately 55 mM (Linsdell, 2017).

#### 4.5.3 Expression profile and contributions to asthma

CFTR is located in the apical membrane of epithelial cells, especially in pancreatic duct and nasal polyps, and to a lesser extent in the intestine, sweat glands, placenta, liver, and predominantly in the male reproductive system, particularly in vas deferens and lungs. Among these, the pulmonary secretory cells are the most crucial in expressing CFTR in this organ. This channel function is to regulate and transport ions essential for the homeostasis of bodily fluids (Harris et al., 1991; Okuda et al., 2021).

In open state, CFTR allows chloride ions to pass through the epithelial tissues at the apical surface, facilitating the cotransport of bicarbonate (HCO_3_^−^). This function directly influences pH of surfaces of epithelial cells and mucus. CFTR has also been reported to have indirect effects on other channels, such as a strong inhibitory effect on ENaCs, as well as modulating other chloride channels like calcium-activated chloride channels (CaCC) and outward rectifying chloride channels (ORCC) (Gentzsch et al., 2010; Gustafsson et al., 2012; Cant et al., 2014).

Mutations or polymorphisms in gene encoding CFTR can lead to disruptions in the homeostatic functioning of various organs, including the lungs. While cystic fibrosis is the most well-known disease associated with mutations in this gene, studies have also linked these mutations to asthma. Individuals who are heterozygous for CFTR mutations but do not develop cystic fibrosis symptoms, known as carriers, have been found to have an increased risk of developing asthma (Miller et al., 2020; Deletang and Taulan-Cadars, 2022).

Non-clinical studies have characterized the involvement of CFTR in asthma, citing data obtained from human airway tissues. These studies demonstrated that chronic exposure to IL-4 and IL-13 increases CFTR activity (Danahay et al., 2002; Galietta et al., 2002). Moreover, this was confirmed in the trachea of mice subjected to allergic inflammation model induced by *Aspergillus fumigatus* extract (Anagnostopoulou et al., 2010).

These findings were supported by Crespo-Lessmann et al. (2021), who conducted a comparative multicenter cross-sectional descriptive study with 100 asthmatic patients, including those with the classic phenotype of asthma, without hypersecretion, and those with severe asthma (with hypersecretion). They observed that a significant number of these patients had polymorphisms in the CFTR gene, and these mutations coincided with more severe asthma and poorer clinical control. This suggests a potential positive correlation between severe asthma and genetic alterations in CFTR.

Despite on these evidences, there are still aspects of the CFTR-asthma correlation that remain not fully elucidated, necessitating further extensive research to validate the evidence in this field. These efforts are aimed at gaining a better understanding of asthma and opening up new perspectives for treatment.

### 4.6 Piezo-type mechanosensitive ion channel component 1 (PIEZO1)

#### 4.6.1 Molecular structure

Discovered a few years ago, PIEZO1 is a non-selective and mechanosensitive cation channel, which operates as a mechanical transducer, being controlled by the tension in the cell membrane. This channel has a unique and differentiated structure in relation to other ion channels, being characterized by a trimeric complex, with a structure similar to a helix around the central ionic pore that detects mechanical forces. PIEZO1 is mainly present in the lipid bilayer, and can also be found in the cytoplasm, endoplasmic reticulum and nuclear envelope (Coste et al., 2010; Coste et al., 2012).

**FIGURE 8:**
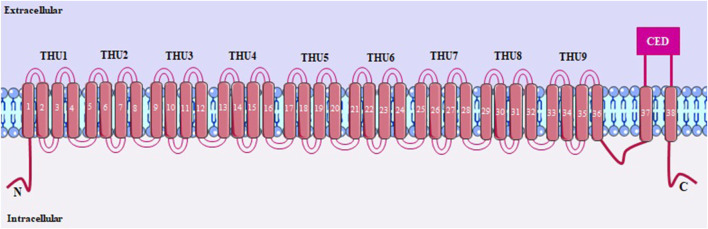
PIEZO1 structure, with 38 transmembrane segments. THUs, transmembrane helical subunits; OH and IH, outer and inner transmembrane helices, respectively; CED, C-terminal domain.

PIEZO1 is a large protein (900 kDa) containing around 2547 amino acids, with each helix having 38 transmembrane segments, which are folded into nine transmembrane helical subunits (THUs), each of which has four segments (Figure 8). The last two transmembrane helices that form the channel pore, the outer (OH) and inner (IH) connected by a C-terminal domain (CED), are fundamental for channel activity, regulating conductance and selectivity (Douguet and Honoré, 2019; Fang et al., 2021; Jiang et al., 2021).

#### 4.6.2 Molecular physiological and biophysical properties

Changes in membrane tension around 1.4 pN nm^−1^, including membrane stretching or compression, shear stress, ultrasound or osmotic pressure can activate PIEZO1 (Bagriantsev et al., 2014), inducing increased conductance to the cations K^+^, Na^+^, Mg^2+^ and, preferably, Ca^2+^ (Volkers et al., 2015). The single-channel conductance for PIEZO1 is 29 pS and the currents turn on in a microsecond scale (<5 msec) with rapid subsequent inactivation (16 msec) (Wu et al., 2016).

#### 4.6.3 Expression profile and contributions to asthma

PIEZO1 is predominant in many types of mechanosensitive cells, such as endothelial, epithelial or immune cells, and is found in organs such as the bladder, skin, kidney, colon and lung (Coste et al., 2010). Several pathophysiological processes involve the participation of PIEZO1, such as blood pressure regulation (Wang et al., 2016), vascular remodeling (Li et al., 2014), red blood cell volume regulation (Cahalan et al., 2015), iron metabolism (Ma et al., 2021), renal fibrosis (He et al., 2022), cancer (Yu and Liao, 2021), diabetes (Zhu et al., 2022), immune cell diapedesis (Offermanns et al., 2021), epithelial homeostasis (Eisenhoffer et al., 2012), regulation of surfactant production (Diem et al., 2020) and lung injury induced by mechanical ventilation (Zhang et al., 2021).

Activation of PIEZO1 through its specific agonist, Yoda1, was found to cause changes in the biomechanics and contractile machinery of airway smooth muscle cells, resulting in relaxation of these cells. These biomechanical behaviors, such as cellular stiffness and traction force, play a key role in cellular functions related to contraction and relaxation. Consequently, the assessment of these parameters in airway smooth muscle is essential to understand the development of hyperresponsiveness in asthma and to identify new therapeutic targets and strategies for the treatment of this disease (Luo et al., 2023).

Another study was performed to determine the role of PIEZO1 in the regulation of adherent junctions in the airway epithelium under positive end-expiratory alveolar pressure (Zhou et al., 2021), which is the result of overinflation due to bronchoconstriction, edema and airflow obstruction that occurs in an asthma attack. The proper functioning of the protective barrier of the airway epithelium depends on the integrity of the adherent junctions (Godfrey, 1997), and damage to these proteins represents damage to the epithelium, which occurs in most respiratory diseases such as asthma (Gon and Hashimoto, 2018).

According to Zhou et al. (2021), the expression of PIEZO1 in bronchial epithelial cells of asthmatic mice in a model induced by ovalbumin was higher compared to healthy animals. Furthermore, treatment with an inhibitor of this channel, GsMTx4, reduced the degradation of adhesion junction proteins, such as occludin, zonula occludens-1 and claudin-18, in primary epithelial cells of human airways subjected to pressure, thus simulating the airways of asthmatic patients *in vitro*. Thus, it was suggested that PIEZO1 plays a crucial role in the degradation of these proteins and compromises the airway epithelial barrier under pressure conditions (Zhou et al., 2021).

### 4.7 Purinergic receptor (P2X)

#### 4.7.1 Molecular structure

P2X receptors are channels that allow the passage of a diversity of ions, such as sodium (Na^+^), calcium (Ca^2+^) and potassium (K^+^), through activation by the presence of extracellular adenosine 5′-triphosphate (ATP). In 1994 the P2X gene was first cloned, thereafter, in 2012, the ATP binding site was discovered, providing information that upon channel activation the ATP-bound state conformation changes (Hattori and Gouaux, 2012; North, 2016; Sheng and Hattori, 2022).

**FIGURE 9:**
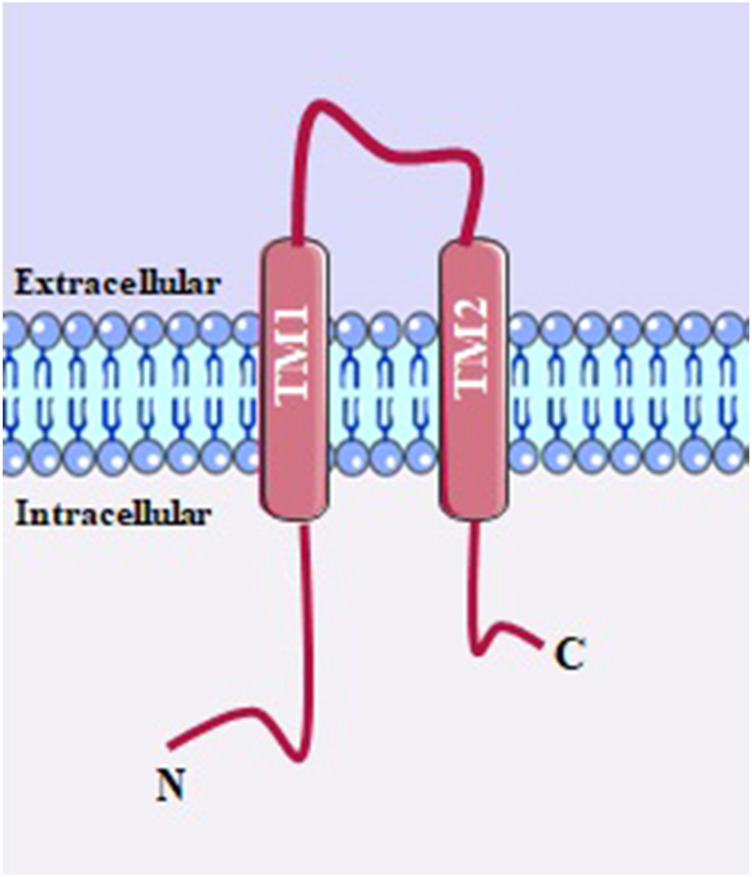
P2X structure with two transmembrane domain α-helices (TM1 and TM2), with TM2 forming the ion-conducting pore within the inner tunnel.

There are seven types of P2X receptors, from P2X1 to P2X7, they exist as either homotrimers or heterotrimers, and when ATP binds to their subunits, it induces a coordinated flexing motion within their extracellular domain and a separation in the transmembrane domain, leading to the opening of a central channel. Each protomer of P2X receptors has a dolphin-like shape, resembling the body, head, dorsal fin, left flipper (LF), and right flipper (RF). The extracellular domain is hydrophilic and contains multiple β-strands. The RF domain has a glycosylation site stabilized by five conserved disulfide bonds. The LF domain, along with the DF domain, plays a crucial role in channel gating. The transmembrane domain consists of two α-helices (TM1 and TM2), with TM2 forming the ion-conducting pore within the inner tunnel (Figure 9) (Burnstock, 2016; Sheng and Hattori, 2022).

#### 4.7.2 Molecular physiological and biophysical properties

P2X receptors exhibit permeability to sodium, potassium, and calcium ions. Some of them additionally demonstrate notable permeability to chloride ions. Quantifying the channel currents of P2X receptors presents challenges due to their rapid flickering nature. Single homomeric P2X2 channels display a unitary conductance of approximately 30 pS and exhibit transient open states that likely correspond to alterations in protein conformation. The permeation properties of the 30-pS state have been extensively studied. Furthermore, during bursts of activity, certain P2X2 channels undergo transitions into a 14-pS state, with some channels exclusively opening to this state (Khakh, 2001; Egan et al., 2006).

#### 4.7.3 Expression profile and contributions to asthma

P2X receptors are widely expressed throughout the body and can be found in both excitatory and non-excitatory cells. They play significant roles in various physiological and pathological processes in mammals, such as pain, inflammation, taste perception, and smooth muscle contraction (Suprernant and North, 2009). In neuroinflammatory conditions observed in both *in vitro* and *in vivo* models, there are reported changes in the expression of P2X receptor subtypes. P2X4 receptors are linked to the early inflammatory mediator, PGE_2_. Similar to P2X7, P2X4 receptors form large conductance pores on the cell membrane, enabling ion efflux and subsequent inflammasome activation. The P2X4 receptor might serve as an initial trigger, while the P2X7 receptor, along with pannexin 1, appears to amplify the signal. In mice, the contribution of the P2X4 receptor to PGE2 release is relatively minor compared to that of P2X7 receptors (Burnstock, 2016).

In asthma, it is already established that P2X receptors play crucial roles in the pathophysiology. Among these, findings suggest that P2X7 receptors could have a significant role in contributing to the undesirable activation of mast cells during chronic inflammatory conditions when there is an increase in extracellular ATP levels (Oguma et al., 2007; Wareham and Seward, 2016). Also, although the molecular mechanism through which P2X4R influences airway remodeling in allergic asthma remains largely unidentified, recent studies suggest that in mice with allergic asthma, the P2X4 receptor (P2X4R) plays a role in airway inflammation and airway remodeling by directly acting on the phenotype switching of BSMCs (Wang, et al., 2018). Another study showed both healthy and asthmatic human eosinophils expressed transcripts for P2X1, P2X4, and P2X5 receptors (Wright et al., 2016).

## 5 Conclusion

**FIGURE 10:**
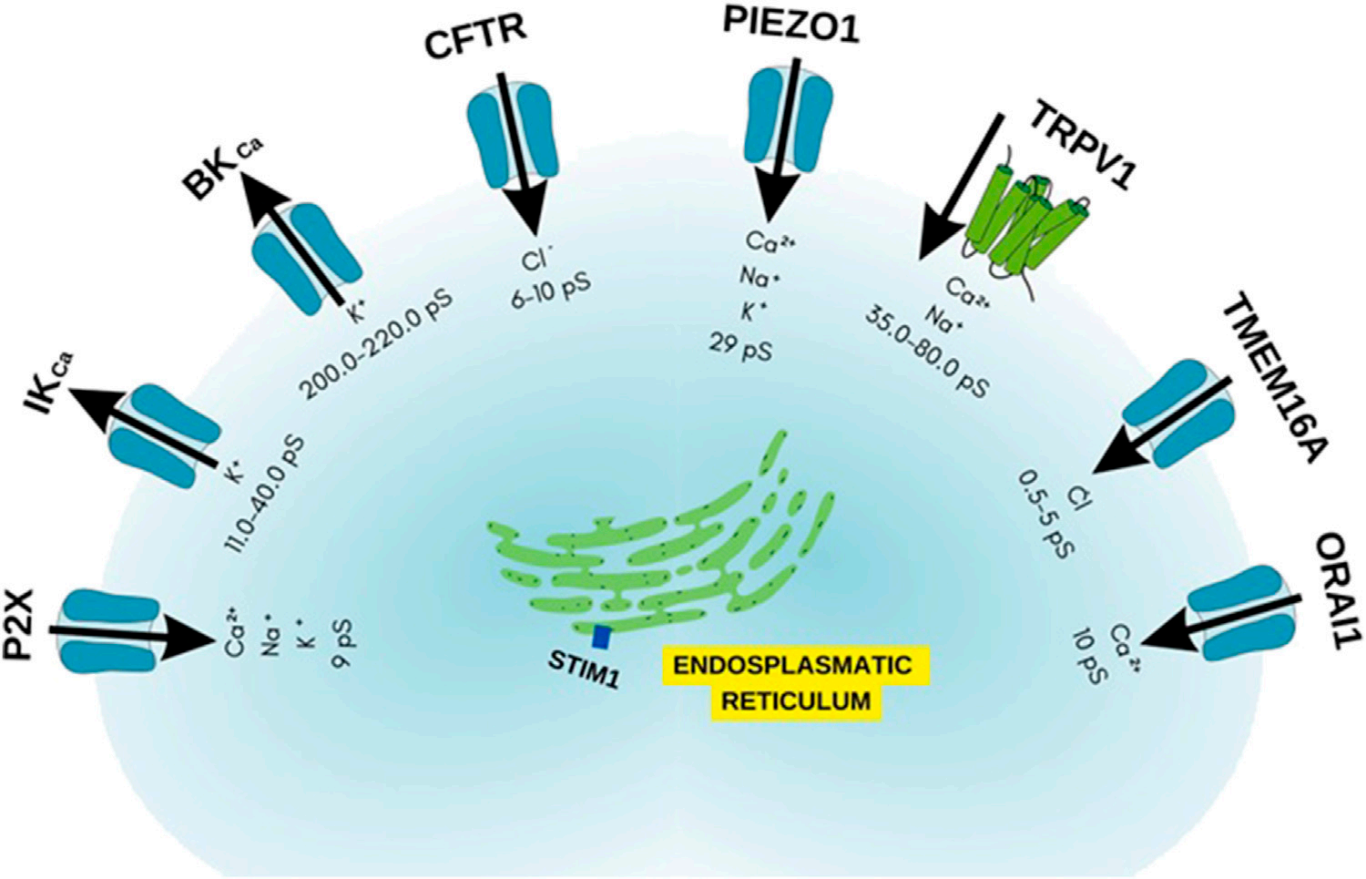
Ion channels as possible targets in allergic asthma.

**FIGURE 11:**
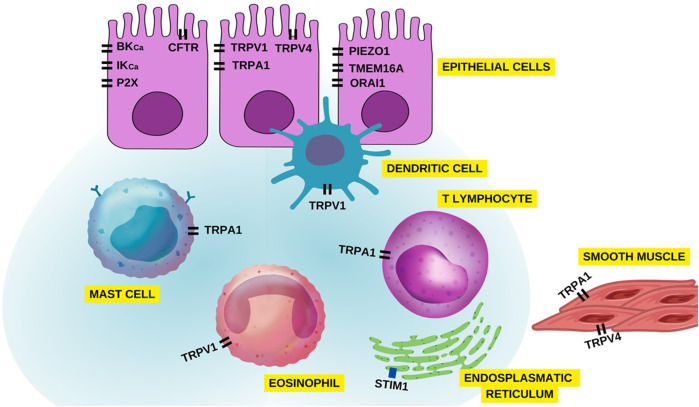
Overview of ion channels in different airway cells associated with asthma pathophysiology.

Based on the studies covered in this review, the importance of ion channels such as transient receptor potential (TRP), stock-operated Ca^2+^ channels (SOCs), Ca^2+^-activated K^+^ channels (IK_Ca_ and BK_Ca_), calcium-activated chloride channel (TMEM16A), cystic fibrosis transmembrane conductance regulator (CFTR), piezo-type mechanosensitive ion channel component 1 (PIEZO1) and purinergic P2X receptor were highlighted in asthma, and these are identified as key elements in the pathogenesis of this disorder (Figure 10). Hence, these channels emerge as potential targets for drug discovery and the development of novel pharmacological tools. Their significance lies in their involvement not only in immune cell function but also in other cell types implicated in asthma, including epithelial cells and airway smooth muscle cells (Figure 11). Nonetheless, additional research is necessary to elucidate the full extent of their promising role in asthma pathophysiology and treatment.

## Figures and Tables

**Table:** 

**APC**	Antigen-presenting cells
**BKCa**	Ca^2+^-activated K^+^ channels of large conductance
**CCL11**	Eotaxin 1
**CCL24**	Eotaxin 2
**CCL26**	Eotaxin 3
**CFTR**	Cystic fibrosis transmembrane conductance regulator
**CRAC**	Ca^2+^ channels activated by Ca^2+^ release
**Cys-LTs**	Cysteinyl-leukotrienes
**DAG**	Diacylglycerol
**DC**	Dendritic cells
**GATA-3**	Type 3 T cell-specific trans-action transcription factor
**GPCR**	G protein-coupled receptors
**IKCa**	Ca^2+^-activated K^+^ channels of intermediate conductance
**IL**	Interleukin
**ILC2**	Type 2 innate lymphoid cells
**MHC**	Histocompatibility complex molecule
**Orai**	Plasma membrane channel pore-forming proteins
**P2X**	Purinergic receptor
**PIEZO1**	Piezo-type mechanosensitive ion channel component 1
**PIP2**	Phosphatidyl inositol 4,5-bisphosphate
**PKA**	Protein kinase A
**PKC**	Protein kinase C
**PLC**	Phospholipase C
**SKCa**	Ca^2+^-activated K^+^ channels of small conductance
**SOC**	Atorage-operated Ca^2+^ channels
**STIM**	Ca^2+^-sensing stromal interacting molecules
**TGF-β**	Transforming growth factor β
**Th**	Type T helper lymphocyte
**TNF**	Tumor necrosis factor
**TRP**	Transient receptor potential
**TRPA**	Transient receptor potential ankyrin
**TRPC**	Transient receptor potential canonical
**TRPM**	Transient receptor potential melastatin
**TRPML**	Transient receptor potential mucolipin
**TRPP**	Transient receptor potential polycystine
**TRPV**	Transient receptor potential vanilloid
**TMEM16A**	Calcium-activated chloride channel
**VEGF**	Vascular endothelial growth factor
